# Structural basis of interaction between the hepatitis C virus p7 channel and its blocker hexamethylene amiloride

**DOI:** 10.1007/s13238-016-0256-7

**Published:** 2016-03-07

**Authors:** Linlin Zhao, Shuqing Wang, Lingyu Du, Jyoti Dev, Liujuan Zhou, Zhijun Liu, James J. Chou, Bo OuYang

**Affiliations:** National Center for Protein Science, State Key Laboratory of Molecular Biology, Shanghai Institute of Biochemistry and Cell Biology, Chinese Academy of Sciences, Shanghai, 200031 China; Tianjin Key Laboratory on Technologies Enabling Development of Clinical Therapeutics and Diagnostics (Theranostics), School of Pharmacy, Tianjin Medical University, Tianjin, 300070 China; Department of Biological Chemistry and Molecular Pharmacology, Harvard Medical School, Boston, MA 02115 USA; Shanghai Science Research Center, Chinese Academy of Sciences, Shanghai, 201204 China

**Dear Editor,**

Hepatitis C virus (HCV) infection, which causes hepatitis C and can chronically lead to serious and life-threatening diseases including liver cirrhosis and hepatocellular carcinoma (Lauer and Walker, 2001), is a rising global health problem. More than 170 million people are infected by HCV worldwide and 3–4 million people are infected each year. No effective vaccines are available to prevent HCV infection. Moreover, HCV is a fast mutating RNA virus with seven distinct genotypes and many subtypes within each genotype. The high degree of genetic diversity can lead to further viral resistance to the current therapies within individual patients (Li et al., 2012). Hence, there remains a strong desire in the medical community to explore new therapeutic opportunities.

p7, the only viroporin encoded by the HCV genome, is a 63-residue protein that oligomerizes in membrane to form ion channels with cation selectivity (Moradpour and Penin, 2013). The p7 channel has been shown to facilitate efficient assembly and release of infectious virions, thus has been sought after as a potential anti-HCV drug target. Several inhibitors have been found to inhibit p7 channel activity with varying efficacies, including the amantadine and rimantadine (Griffin et al., 2003) that also block the influenza M2 channel (Pielak et al., 2011), hexamethylene amiloride (HMA) (Premkumar et al., 2004), and the long-alkyl-chain iminosugar derivatives (Pavlovic et al., 2005). Another novel small molecule known as BIT225, which has partial structural resemblance to HMA, also inhibits p7 activity and has been pursued as an anti-HCV drug candidate in clinical trials (Luscombe et al., 2010).

For many years, the inhibition mechanism of the known p7 inhibitors remained elusive in the absence of a high-resolution p7 structure. Previous solution NMR studies revealed that the adamantane compounds bind to six equivalent hydrophobic pockets (due to the 6-fold symmetry of the p7 hexamer) near the kink of the central pore-forming helices, consisting of elements from different helical segments and from different subunits (OuYang et al., 2013). A more recent study showed that insertion of rimantadine into these pockets could function as “molecular wedge” that attenuates conformational breathing needed for cation flow through the N-terminal constriction of the p7 channel (Dev et al., 2015). In addition to the studies on the p7 hexamer, a combined NMR and modeling study modeled the binding of rimantadine and several new compounds in the context of a p7 heptamer complex (Foster et al., 2014). Compared to rimantadine, HMA has a completely different chemical structure and was reported to inhibit p7 channel in different genotypes with stronger inhibition at similar drug concentration (Griffin et al., 2008). Earlier NMR titrations showed that HMA caused larger chemical shift perturbations in the loop region, while amantadine and NN-DNJ have a larger effect on the chemical shifts of the terminal regions (Cook et al., 2013). Therefore, HMA may act on a different site of p7 than rimantadine, and it is of substantial interest to identify this binding site.

Here, we capitalized on the NMR system of the p7 channel from genotype 5a, designated p7 (5a) and used a combination of NMR titration and nuclear Overhauser enhancement (NOE) experiments to characterize HMA binding to the p7 channel. We used 100 µmol/L p7 (5a) (monomer concentration) solubilized in 20 mmol/L DPC and recorded good quality ^1^H-^13^C HSQC spectra of the methyl groups and ^1^H-^15^N TROSY-HSQC spectra of the backbone amine at various drug concentrations. NMR titration experiment showed that titrating the p7 channel with HMA had no effects on the methyl groups of Val25 and Val26 (Fig. 1A), which are key constituents of the rimantadine binding pocket (OuYang et al., 2013). In contrast, rimantadine titration strongly perturbed the Val25 resonance (Fig. 1A). Remarkably, HMA titration induced chemical shift changes at the N-terminal constriction of the channel (marked by Val5 and Ile6), very similar to those caused by rimantadine binding. The methyl HSQC spectra show that the two different compounds perturbed the Cγ_1_H_3_ of Val5 (Fig. 1B) and the Cδ_1_H_3_ of Ile6 (Fig. 1C) with essentially the same pattern. Moreover, the overall chemical shift change patterns in the ^1^H-^15^N TROSY-HSQC spectra induced by HMA and rimantadine are also very similar (Fig. S1).

**Figure 1:**
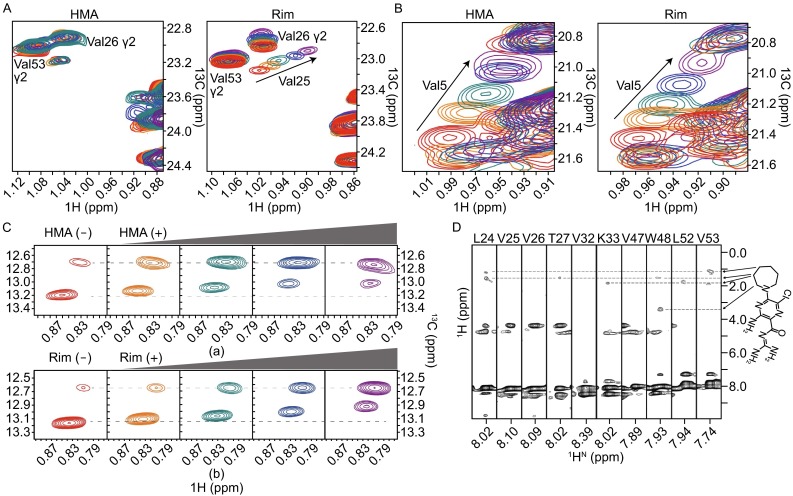
**NMR characterization of HMA binding site**. (A–C) Rimantadine and HMA titrations using ^1^H-^13^C HSQC as a readout. Uniformly (^13^C, ^15^N)-labeled p7 (5a) (monomer concentration at 0.1 mmol/L) reconstituted in 20 mmol/L DPC was titrated with 0 (red), 1 (orange), 2 (green), 4 (blue), and 8 mmol/L (purple) rimantadine and HMA. Spectra were recorded on a 600 MHz spectrometer. The resolved peaks are labeled with assignments and the arrows indicate the movement of peaks. Shown in the boxes are gamma methyl resonances of Val25 (A), gamma methyl resonances of Val5 (B), and delta methyl resonances of Ile6 (C). (D) NOESY experiment identifying the binding site. Representative strips from the three-dimensional ^15^N-edited NOESY-TROSY-HSQC spectrum (300 ms NOE mixing time) recorded using a sample containing (^2^H,^15^N)-labeled p7 (5a) and 2 mmol/L HMA, showing HMA NOEs to the backbone amide protons of Leu24, Thr27, Lys33, Trp48, Leu52, and Val53

We then performed more direct experiments to accurately pinpoint the HMA binding site by measuring protein-drug NOEs. Detection of HMA-specific NOEs was made possible by using a sample that contains (^15^N, ^2^H)-labeled p7 (5a) and deuterated or “NMR invisible” DPC. The 3D ^15^N-edited NOESY-TROSY-HSQC spectrum of this sample showed strong HMA NOE crosspeaks corresponding to the hexamethylene for Leu24, Trp48, Leu52, and Val53 and weaker hexamethylene NOE crosspeaks for Thr27 and Lys33 (Fig. 1D). Our earlier NMR investigation of rimantadine binding to p7 (5a) observed NOE crosspeaks between the adamantane protons and the amide protons of Val26, Leu56, and Arg57 (OuYang et al., 2013). The differences in intermolecular NOE patterns between HMA and rimantadine are consistent with the differences in NMR titration data between the two inhibitors shown in Fig. 1A. Altogether, the NMR data indicate that HMA binds to a different site compared to the one that adamantane compounds bind to. We could not detect any NOEs to the NH_2_ groups of HMA due to fast solvent exchange, and thus the orientation of the bound HMA could not be determined using the NOE data.

MD simulation was subsequently used to more accurately position and orient the HMA in the NMR-derived binding site by optimizing physical interactions. Six HMA molecules were placed in the 14 × 14 × 14 Å^3^ box containing the p7 (5a) channel embedded in neutral POPC bilayer such that the position of the hexamethylene moiety of each of the HMAs satisfies the observed intermolecular NOEs. After 50 ns of MD simulation, the positions of HMAs did not change significantly, although the orientation became more defined (Fig. 2A–C). The hydrophobic hexamethylene ring binds to the above-mentioned pocket consisting of Trp48, Leu52, and Val53 from H3 of the i monomer, Lys 33 from H2 of the i + 1 monomer, and Leu24 and Thr27 from H2 of i + 2 monomer (Fig. 2D), and the position of hexamethylene overall agrees with the NOE data. The monitored distances between the heavy atoms (carbons and nitrogens) during the simulation are approximately equal to the NOE-derived distances (~4 Å) plus 2 Å. The amiloride moiety of HMA points away from the bilayer core into the solvent exposed region near the wider opening of the channel cavity (Fig. 2D). The structural data of HMA binding to p7 (5a) allow for modeling the HMA binding site in p7 (1a), for which HMA inhibition of the channel activity has been experimentally shown (Premkumar et al., 2004). Based on the sequence alignment (Fig. S2A), we used the NMR structure of p7 (5a) (PDB ID: 2M6X) as a template and developed a 3D structural model of p7 (1a) via Discovery Studio 4.0 (DassaultSystèmesBIOVIA, 2015) by “segment matching” and “coordinate reconstruction” approach (Fig. S2B). As in the case of p7 (5a), we performed 50 ns of MD simulation using the p7 (1a) model. The results show that p7 (1a) forms a hydrophobic binding pocket for HMA very similar to that observed in p7 (5a) (Fig. S2C).

**Figure 2:**
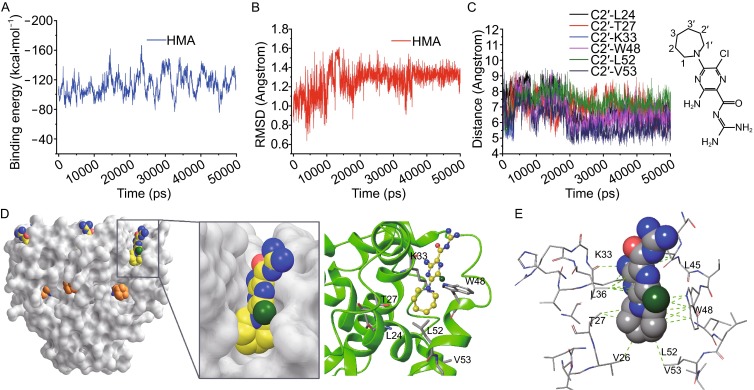
**MD simulation refinement of HMA binding site**. (A) The binding energy between HMA and other surrounding materials in complex system. (B) RMSD value of heavy atoms between the sampled conformations and the starting conformation of HMA during the 50 ns MD production simulation. (C) The distances between the three key carbons of hexamethylene ring of HMA (the three key carbons, C1, C2, and C3, were indicated in HMA structure) and the backbone N atoms of residues L24, K33, W48, L52, and V53 were monitored during the 50 ns MD production simulation. (The monitored distances between carbon and N atoms are approximately equal to the NOEs data plus 2 Å). (D) Comparison between adamantane binding site and HMA binding site of HCV p7 channels. Left panel: The peripheral pockets that wrap around the adamantane cage of rimantadine (orange) and the hexamethylene group of HMA (yellow). Right panel: A representative pocket of HMA binding among six equivalent pockets in the p7 hexamer. (E) The MD simulation result showing the hydrophobic interactions and the polar interactions between HMA and p7 (5a)

Our NMR and MD results show that HMA and rimantadine bind to different but nearby peripheral pockets of the p7 channel. Although the NMR data was collected for the p7 (5a), the high sequence identity between 5a and 1a and the fact that the p7 (5a) residues involved in HMA binding are mostly conserved in p7 (1a) provided us with strong confidence that the HMA binding site reported in this study is relevant to the identified HMA inhibition and likely applicable to other p7 variants. The binding of HMA and rimantadine both involve anchoring of their hydrophobic groups to a hydrophobic pocket adjacent to the channel cavity. In both cases, the binding site is not inside the channel cavity and thus there are six equivalent binding sites due to the six-fold symmetry of the p7 channel. Overall, HMA binding can be characterized by insertion of the relatively flat HMA molecule into a crevice near the wider mouth of the channel cavity where the hydrophobic hexamethylene interacts with the hydrophobic pocket deep inside the crevice and the polar amiloride fills the solvent accessible portion of the crevice (Fig. 2D). We note that previous studies of drug binding of p7 using a heptameric hairpin structure model yielded different peripheral binding sites for inhibitors. The differences between the two models might relate to the genotypes and sample environment. However, some key residues are conserved in both models, for example, Trp48 and Leu52 (Foster et al., 2014).

Although different binding sites, NMR chemical shift perturbation data in Fig. 1A–C indicate that the two inhibitors induce very similar long-range conformational changes near the narrow opening of the cavity. For both inhibitors, the binding site is far away from the narrow end of the cavity. In fact, compared to the rimantadine binding site, the HMA site is even farther away. We thus hypothesize that the mechanism of inhibition by HMA is also allosteric, either by restricting movement of helices needed for ion flow or by inducing structural rearrangements that block ion conduction, or both.

It is interesting to note that the HMA and rimantadine binding sites both contain several hydrophobic residues and the two residues they have in common are Leu52 and Val53. Mutations of L(50–55)A that decreased hydrophobicity have been shown to result in the loss of rimantadine sensitivity, and other observed mutation with decreased hydrophobicity also confers drug resistance in p7 (Foster et al., 2011). Since the known inhibitors of p7 channel, although structurally very different, contain a hydrophobic component, we believe one of the key determinants of p7 allosteric inhibitors is hydrophobic interaction. In the case of HMA, besides the hydrophobic interactions of hexamethylene with Leu24, Leu52 and Val53, the amiloride of HMA forms hydrogen bond with Lys33 and interacts with Leu36 and Leu45 by Van der Waals force (Fig. 2E). The extra polar interactions between HMA and p7 may account for the stronger inhibition. Further functional validation of HMA binding would be highly desirable to validate these interactions to provide a future direction for structure-guided inhibitor design.

Finally, obtaining structural information of weak inhibitors binding to ion channels is notoriously difficult. The combined use of NMR to identify the proximal ligand binding site and MD simulation to refine the channel-ligand complex as demonstrated here for the HCV p7 channel represents an effective solution to harvest useful information from many known weak inhibitors of ion channels.

## Electronic supplementary material

Below is the link to the electronic supplementary material.

Supplementary material 1 (PDF 542 kb)
