# Supplementary material for: Structural basis of interaction between the hepatitis C virus p7 channel and its blocker hexamethylene amiloride
Source: Protein Cell. 2016 Mar 7;7(4):300–4. doi: 10.1007/s13238-016-0256-7 (PMC4818843; doi:10.1007/s13238-016-0256-7)

Supplemental Materials

Protein expression and purification

The p7 (5a) protein was prepared as previously described (OuYang et al., 2013). Briefly, p7 was transformed in *E. Coli* BL21 (DE3) cells as a fusion to His9-trpLE in the pMM-LR6 vector and grown in M9 minimal medium. Inclusion bodies were extracted and solubilized in 6 M guanidine HCl, 50 mM Tris (pH 8.0), 200 mM NaCl, 1% Triton X-100 (Buffer A). The His9-trpLE-p7 fusion protein was first purified by Ni^{2+} affinity chromatography in Buffer A at room temperature and eluted from the Ni^{2+} column in the same buffer with 400 mM imidazole. The eluted protein was cleaved at the methionine position by cyanogen bromide (CNBr) in 80% formic acid. The p7 and fusion protein were then separated by reverse-phase chromatography using a C18 column (Proto). NMR samples were prepared by dissolving 1-2 mg of lyophilized p7 peptide in 6 M guanidine HCl and 20 mg dodecylphosphocholine (DPC) and refolded by dialysis against the NMR buffer (25 mM MES, pH 6.5).

NMR titration experiments for p7 (5a) oligomer

All NMR based small molecule titration experiments were recorded at 30 ^{o}C using Bruker 600 MHz spectrometer equipped with cryogenic probe. Rimantadine and HMA were dissolved in dimethyl sulphoxide (DMSO) to make 200 mM stock solutions, which were subsequently added to 0.1 mM (^{15}N, ^{13}C)-labeled p7 (5a) sample reconstituted in 20 mM DPC micelles. Two-dimensional ^{1}H-^{15}N TROSY-HSQC and ^{1}H-^{13}C HSQC (optimized for methyl groups) were recorded at each of the inhibitor concentrations including 0, 1, 2, 4, and 8 mM. The chemical shift changes at

each inhibitor concentration were analyzed using the CcpNmr software (Vranken et al., 2005).

NMR NOE experiments for protein–inhibitor interaction

The nuclear Overhauser enhancement (NOE) experiment for assigning intermolecular contacts between the p7 (5a) channel and HMA was performed at 30 ^{o}C using Bruker 900 MHz spectrometer equipped with cryogenic probe. We used a sample system in which p7 (5a) is (^{2}H, ^{15}N)-labeled and DPC is deuterated to enable exclusive detection of intermolecular NOEs between the protein backbone amide protons and the non-exchangeable HMA protons using the three-dimensional ^{15}N-edited NOESY-TROSY-HSQC experiment (Xia et al., 2000). The sample used for this experiment contained 0.8 mM p7 (5a) (monomer), 160 mM deuterated DPC, and 2 mM HMA. The NOE mixing time used was 300 msec. The NMR spectra were processed using NMRPipe (Delaglio et al., 1995) and analyzed using XEASY (Bartels et al., 1995).

Molecular dynamics simulation

The MD simulations were performed using the Desmond 3.6 program package (Bowers et al., 2006) and the OPLS-AA 2005 force field (Peng and Kaminski, 2005) in a neutral 1-palmitoyl-2-oleoyl-sn-glycero-3-phospcholine (POPC) bilayer with appropriate number of counter ions to balance the net charge of the system solvated in 0.15 M NaCl. The membrane localization of the p7 (5a) hexameric complex was defined using the Orientations of Proteins in Membranes (OPM) database (Lomize et al., 2006). The protein and ligand molecules in POPC bilayer were contained in a

periodic box (14×14×14 Å^3) containing the explicit simple point charge (SPC) waters. Nose-Hoover temperature coupling (Hoover, 1985) and Martina-Tobias-Klein method (Martyna, 1994) with isotropic scaling (Hoover et al., 1985) were used to control the simulation temperature (310 K) and atmospheric pressure (1 atm). The particle-mesh Ewald (PME) method (Cerutti et al., 2009; Shan et al., 2005) was used to calculate long-range electrostatic interactions with grid spacing of 0.8 Å. Van der Waals (VDW) and short-range electrostatic interactions were smoothly truncated at 9.0 Å. A RESPA integrator (Deng et al., 1992) was used and the real-space parts of electrostatic and VDW interactions were cut off at 9 Å. Before MD simulations, the system was equilibrated using the default membrane relax protocol provided in Desmond, which consists of a series of restrained minimizations and MD simulations that are designed to slowly relax the system without deviating too much from the initial protein coordinates. The initial p7 (5a) channel coordinates for the MD calculations were taken from PDB ID 2M6X. After removing all geometry restraints (bonds, angles, planarity, chirality, dihedral and etc.) used in refinement via the 2 ns system minimization and relaxation, the system was subject to 50 ns of normal pressure and temperature (NPT) production simulation during which the configuration was saved every 4 ps. The structure model of p7 (1a) was built via Discovery Studio 4.0 (DassaultSystèmesBIOVIA, 2015) by “segment matching” and “coordinate reconstruction” approach. The MD simulation of p7 (1a) was performed following the same procedure as p7 (5a).

Supplemental References

Bartels, C., Xia, T.H., Billeter, M., Guntert, P., and Wuthrich, K. (1995). The program XEASY for computer-supported NMR spectral analysis of biological macromolecules. Journal of biomolecular NMR 6, 1-10.

Bowers, K.J., Dror, R.O., and Shaw, D.E. (2006). The midpoint method for parallelization of particle simulations. The Journal of chemical physics 124, 184109.

Cerutti, D.S., Duke, R.E., Darden, T.A., and Lybrand, T.P. (2009). Staggered Mesh Ewald: An extension of the Smooth Particle-Mesh Ewald method adding great versatility. Journal of chemical theory and computation 5, 2322.

DassaultSystèmesBIOVIA (2015). Discovery Studio Modeling Environment (San Diego, Dassault Systèmes).

Delaglio, F., Grzesiek, S., Vuister, G.W., Zhu, G., Pfeifer, J., and Bax, A. (1995). NMRPipe: a multidimensional spectral processing system based on UNIX pipes. Journal of biomolecular NMR 6, 277-293.

Deng, Z., Martyna, G.J., and Klein, M.L. (1992). Structure and dynamics of bipolarons in liquid ammonia. Physical review letters 68, 2496-2499.

Hoover, W.G. (1985). Canonical dynamics: Equilibrium phase-space distributions. Physical review A 31, 1695-1697.

Hoover, W.G., Ciccotti, G., Paolini, G., and Massobrio, C. (1985). Lennard-Jones triple-point conductivity via weak external fields: Additional calculations. Physical review A 32, 3765-3767.

Lomize, M.A., Lomize, A.L., Pogozheva, I.D., and Mosberg, H.I. (2006). OPM: orientations of proteins in membranes database. Bioinformatics 22, 623-625.

Martyna, G.J. (1994). Remarks on "Constant-temperature molecular dynamics with momentum conservation". Physical review E, Statistical physics, plasmas, fluids, and related interdisciplinary topics 50, 3234-3236.

OuYang, B., Xie, S., Berardi, M.J., Zhao, X., Dev, J., Yu, W., Sun, B., and Chou, J.J. (2013). Unusual architecture of the p7 channel from hepatitis C virus. Nature 498, 521-525.

Peng, Y., and Kaminski, G.A. (2005). Accurate determination of pyridine-

poly(amidoamine) dendrimer absolute binding constants with the OPLS-AA force field and direct integration of radial distribution functions. The journal of physical chemistry B 109, 15145-15149.

Shan, Y., Klepeis, J.L., Eastwood, M.P., Dror, R.O., and Shaw, D.E. (2005). Gaussian split Ewald: A fast Ewald mesh method for molecular simulation. The Journal of chemical physics 122, 54101.

Vranken, W.F., Boucher, W., Stevens, T.J., Fogh, R.H., Pajon, A., Llinas, M., Ulrich, E.L., Markley, J.L., Ionides, J., and Laue, E.D. (2005). The CCPN data model for NMR spectroscopy: development of a software pipeline. Proteins 59, 687-696.

Xia, Y., Sze, K., and Zhu, G. (2000). Transverse relaxation optimized 3D and 4D 15n/15N separated NOESY experiments of 15N labeled proteins. Journal of biomolecular NMR 18, 261-268.

Supporting Figure Legends

Figure S1. Chemical shift perturbations of 15N-labeled p7 (5a) (monomer concentration at 0.5 mM) reconstituted in 100 mM DPC in the presence of in the presence of 4 mM HMA (black) and 4 mM rimantadine (blue). Spectra were recorded on a 600 MHz spectrometer.

Figure S2. (A) Sequence alignment between p7 (5a) and p7 (1a) for showing the conserved residues between the two genotypes. Residues strictly conserved in both 1a and 5a are highlighted in yellow; partially conserved are highlighted in cyan. The six residues involved in HMA binding are colored in red. (B) The overlayed structures of p7 (1a) in red and p7 (5a) in green. The structure model of p7 (1a) is built using the PDB ID 2M6X of p7 (5a). (C) The MD simulation result showing the HMA binding site of p7 (1a).

Figure S1

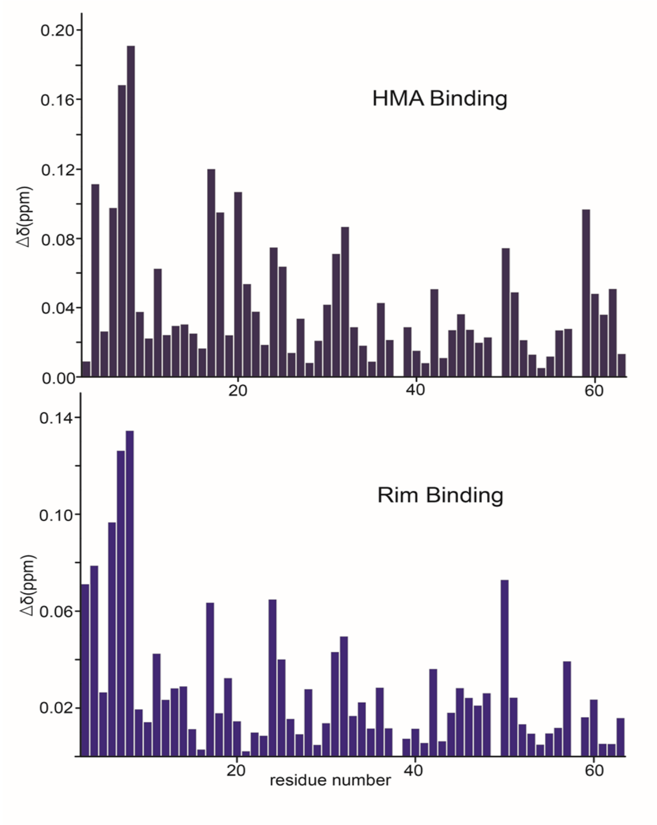

Figure S2

A

```
Genotype 1          10        20         30        40         50        60
5a       GAKNVIVLNAASAAGNHGFFWGLLVVTLAWHVKGRLVPGATYLSLGVWPLLLVRLLRPHRALA
1a       ALENLVILNAASLAGTHGLVSFLVFFCFAWYLKGRWVPGAVYAFYGMWPLLLLLLALPQRAYA
```

B

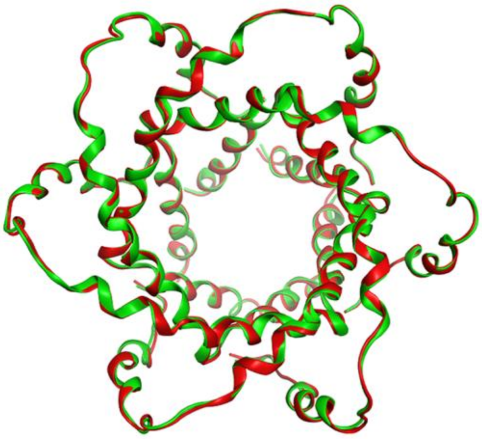

C

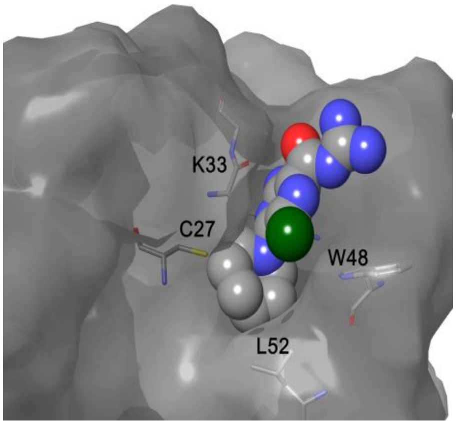

Supplement: Supplementary file 1 — Supplementary material 1 (PDF 542 kb) [file 13238_2016_256_MOESM1_ESM.pdf]
